# Understanding the Relationship between Predictors of Alcohol Consumption in Pregnancy: Towards Effective Prevention of FASD

**DOI:** 10.3390/ijerph17041388

**Published:** 2020-02-21

**Authors:** Isabel Corrales-Gutierrez, Ramon Mendoza, Diego Gomez-Baya, Fatima Leon-Larios

**Affiliations:** 1 Foetal Medicine Unit, University Hospital Virgen Macarena, C.P. 41009 Seville, Spain; icorrales@us.es; 2 Department of Surgery, University of Seville, 41009 Seville, Spain; 3 Department of Social, Developmental and Educational Psychology, University of Huelva, 21007 Huelva, Spain; ramon@dpsi.uhu.es; 4 Research Group on Health Promotion and Development of Lifestyle across Lifespan, University of Huelva, 21007 Huelva, Spain; 5 Center for Research in Contemporary Thought and Innovation for Social Development (COIDESO), 21007 Huelva, Spain; 6 Nursing Department, Faculty of Nursing, Physiotherapy and Podiatry, University of Seville, 41009 Seville, Spain; fatimaleon@us.es

**Keywords:** prevention, alcohol consumption, pregnancy, FASD, lifestyle, public health, Spain

## Abstract

Background: Prenatal alcohol exposure can produce serious changes in neurodevelopment that last a lifetime, as well as a wide range of congenital abnormalities, and is the main non-hereditary, avoidable cause of intellectual disability in developed countries. It is therefore crucial to understand the determinants of alcohol consumption during pregnancy. This study is aimed at determining the factors that predict it, as well as the interactions between them. Methods: A cross-sectional study was carried out using a random sample of 426 pregnant women being treated at the outpatient clinic of a public university hospital in Seville (Spain), when they were in their twentieth week of pregnancy. A custom-designed questionnaire was used for data collection and applied in the course of an interview administered by trained health professionals. The data collected were analyzed using hierarchical regression, moderation analysis, and a structural equations model. Results: Alcohol consumption prior to pregnancy proved to be the most powerful predictor of alcohol intake during pregnancy. Other particularly significant predictors were the percentage of professionals who gave correct advice to the expectant mother—not to consume any alcohol during pregnancy—and perception of the risk from drinking wine during pregnancy. The number of pregnancies correlates positively with alcohol intake during pregnancy, while the expectant mother’s level of education correlates negatively. Conclusions: Identifying these predictive factors will allow the design of more effective fetal alcohol spectrum disorder (FASD) prevention strategies.

## 1. Introduction

Currently, there is extensive evidence of the teratogenic effects of prenatal alcohol exposure, which can translate into a broad spectrum of abnormalities that make up what is known as fetal alcohol spectrum disorder (FASD) [1,2]. The damage caused to the forming nervous system is permanent and has a number of consequences for the biological development of the fetus as a whole, as well as for subsequent neurocognitive and social development in childhood and the other stages in lifespan [3]. FASD therefore represents a major health and socio-educational problem, since it is the main non-hereditary, avoidable cause of learning difficulties in developed countries, with serious consequences for new-born babies that will last their entire lives [4,5,6]. 

It is uncertain how prevalent alcohol consumption during pregnancy is, due to the tendency for it to be underestimated when it is assessed using scales, and as a result of the lack of studies with biomarkers that allow it to be estimated reliably. However, there are worrying data available that tell us that alcohol consumption is a very widespread practice among expectant mothers (particularly in Europe, North America, Australia, and in countries such as South Africa). Its incidence worldwide has been estimated at 9.8% [7]. In a study carried out on expectant and new mothers found online from eleven European countries, 15.8% of them stated that they consumed alcohol during pregnancy, with the United Kingdom (28.5%) and Russia (26.5%) being the countries with the highest estimated level of prevalence in this study [8]. In parallel to consumption, the countries that have the highest estimated prevalence of FASD (19.8 per 1000 population) are those that belong to the European region of the WHO [9]. All of this makes FASD a global public health problem, requiring effective strategies for prevention and early diagnosis, and representing a crucial challenge for healthcare personnel in general, and obstetricians and midwives in particular. 

Health professionals play a very important role in providing preventive advice regarding healthy lifestyles in the periconceptional period, during pregnancy, and postnatally. However, there are signs that large sections of healthcare professionals (general practitioners (GPs), obstetricians, and midwives) are not doing their job fully and properly in this regard. The health advice that pregnant women receive about the risks inherent in consuming alcohol during pregnancy frequently proves contradictory, or else it does not reach those with a lower level of education effectively [10]. A number of studies suggest that not having received any specific training in this area could explain why not all professionals routinely inquire about alcohol consumption when caring for pregnant women, or do not always provide appropriate information on the subject [11,12].

Another factor which might explain this is the ambivalence of the official guidelines themselves in this area, or their inconsistency from one country to another, and over time. A review of the Australian and American guidelines related to alcohol consumption during pregnancy, carried out by Whitehall in 2006 [13], highlighted the fact that there was a certain permissiveness shown by the health authorities regarding alcohol consumption during pregnancy. They even failed to recommend abstention, arguing that it might cause disproportionate anxiety and therefore prove even more harmful than alcohol consumption. At the same time, another review of the policies and guidelines on alcohol consumption during pregnancy in English-speaking countries [14] showed that these varied from country to country and within the countries themselves. With the passing of time, since it has not been possible to establish that there are safe levels of alcohol consumption during pregnancy, and at the same time, since there is increasing evidence that low levels of alcohol consumption can lead to risks to fetal development [15], there are many countries where official health guidelines on pregnancy recommend abstaining completely from drinking alcohol during this time. Thus, since 2002, France has recommended total abstention from alcoholic beverages during pregnancy [16]. National public health bodies in Australia [17], Denmark [18], and Norway [19] have made equivalent recommendations. At the same time, in Scotland, since 2012, the Chief Medical Officer has advised that “pregnant women and those trying to conceive should avoid alcohol” [20]. Equally, in the USA, the Message to Women from the U.S. Surgeon General stated “No amount of alcohol consumption can be considered safe during pregnancy” [21]. At the same time, the American Academy of Pediatrics recommends that health professionals promote total avoidance of alcohol consumption throughout pregnancy, in line with the principle of precaution [4]. In Canada, the 2010 consensus guidelines from the Society of Obstetricians and Gynaecologists of Canada support alcohol abstention during pregnancy [22].

In order to be able to develop effective FASD prevention, the starting point must be a good understanding of the current situation of the problem. It is particularly crucial to know about the factors that cause or encourage expectant mothers to consume alcohol during pregnancy.

There are some studies which aim to identify the factors that predict alcohol consumption during pregnancy. Several of them have concluded that the most important factor is the frequent consumption of alcohol prior to pregnancy [23,24,25,26,27]. Bearing this in mind, it is particularly worrying that in countries such as Spain, at present, two thirds of women of child-bearing age consume alcohol. According to the Spanish National Health Survey of 2017 [28], only 36.96% of women aged 15–24 years of age and 23.78% aged between 25 and 34 identified themselves as non-drinkers. These data present us with a potential problem in the years to come—an increased incidence of FASD—unless effective healthcare provided during the periconceptional period helps them to stop consuming alcohol.

Other predictive factors identified in some studies are having been the target of violence [25], high socioeconomic status, an unplanned pregnancy, and late childbearing age [29]. Similarly, smoking or using other drugs prior to becoming pregnant prove to be predictors of alcohol consumption during pregnancy [26,30,31].

Research to date into the factors that predict alcohol consumption during pregnancy is limited and some yield contradictory results. There are also some potential predictors that have scarcely been explored, such as obstetric history, the partner’s alcohol consumption, health advice received regarding alcohol consumption during pregnancy, and the perception of damage resulting from prenatal exposure to alcohol. These factors, in real life, presumably do not act in isolation, but rather interact with one another. To our knowledge, the interaction between a wide range of predictive factors of alcohol consumption during pregnancy has not been studied to date. It is also particularly important to identify these predictors and the interactions between them in those regions of the world where there is a combination of a high rate of alcohol consumption among women of childbearing age and limited implementation of healthcare programs aimed at FASD prevention. The case of Spain, just like that of other European countries, may be particularly illustrative for all regions of the world where these circumstances exist.

Therefore, our study, conducted using a sample of expectant mothers who attended a routine pregnancy check-up in a city in the south of Europe, aimed to determine a wide range of factors that predict alcohol consumption during pregnancy and to identify the relative weight of each of them. Additionally, the study was also designed to assess the degree of interaction between different factors (sociodemographic factors, obstetric history, the partner’s alcohol consumption, health advice received, and beliefs about the possible risks) and how much they moderate the relationship between previous consumption and alcohol intake during pregnancy.

## 2. Participants and Methods

### 2.1. Study Design

A cross-sectional study was carried out, through interviews, on a representative sample of the pregnant women treated in a publicly managed university hospital in Seville (Spain). The sample was randomly selected from women who attended the morphology ultrasound clinic, located in the outpatient area of the hospital, in their 20th week of pregnancy, during a five-month period in 2016.

### 2.2. Data Collection and Participants

The population of expectant mothers in the twentieth week of pregnancy in the health area of this university hospital during the period when the data were collected was 1664. The sample selection criteria were set as an interview with one out of every two pregnant women, to be chosen at random, i.e., 832 pregnant women. Of these, 426 agreed to be interviewed. The minimum desired sample size was 400 participants. All of them had the same gestational age, and in this regard, it was a homogeneous sample and one that was representative of the population of pregnant women treated by the aforementioned public hospital. For the collection of data, face-to-face interviews were conducted, carried out by health professionals who had previously been instructed on how to do so. The questionnaire was custom designed by the research group and was delivered under conditions that ensured the anonymity of those interviewed and the confidentiality of the information collected. 

The eligibility criteria for inclusion in the study were: Pregnant women of 16 years of age or older, who speak and read Spanish fluently, who accepted and signed the informed consent for inclusion in the study. Further characteristics of the sample are described elsewhere [32].

### 2.3. Ethics

Before the study was carried out, both its protocol and the questionnaire prepared by the research group were approved by the Clinical Research Ethics Committee of the University Hospital Virgen Macarena (Research code: ICG15/Internal code: 0254N-15). 

As a prerequisite for conducting the interview, pregnant women were given oral and written information about the study and an informed consent form, which they had to complete and sign voluntarily if they wanted to be part of the study, delivering one copy to the research team and keeping the other. This documentation reflected all the information related to the objective of the study, as well as the guarantees of confidentiality, privacy, and preservation of anonymity in the responses. The participants gave their informed consent by signing and returning this form. The Helsinki declaration of 1975 and its subsequent amendments were respected.

### 2.4. Questionnaire

The instrument used for the collection of information was a questionnaire prepared and designed ad hoc for the study by the team of researchers who conceived and carried out this research project. Each of the completed questionnaires was given a code that preserved the anonymity of the users. The members of the research group included health professionals (a GP, two from the field of obstetrics, in particular—an obstetrician and a midwife—and a neonatologist), as well as professionals from the fields of psychology and sociology. The experience of all of them, as well as their knowledge in the field as a result of their research background, made it possible to prepare the customized questionnaire for the population to which it was addressed. Furthermore, a preliminary pilot was carried out in order to verify understanding of the questions, as well as the possibility of adding or removing categories in the answers to the multiple-choice questions. The questions regarding consumption patterns were taken from the Alcohol Use Disorders Identification Test (AUDIT) [33]. 

Most of the questions in the questionnaire provided the possibility of answering with several predetermined options; however, one of them was “other”, which allowed the interviewer to note down all answers provided spontaneously by the pregnant women that were not in line with the categories established. Subsequently, the research group transcribed these answers to categorize them into the options that had been established beforehand, or put them into a new category, thus preventing information from being lost. In addition to these multiple-choice questions, there were also open-ended questions in the questionnaire that were recorded by taking notes. After the data were collected, categories were created for these answers based on a thematic analysis of them.

The questionnaire’s content covers the following groups of variables:

(a) Sociodemographic variables: Age, educational level (categorized into three groups from lowest to highest level: (1) Low level of studies, e.g., primary education; (2) medium level of studies, e.g., compulsory secondary education, professional training; (3) university studies and employment status (categorized from the best to the worst employment status in five groups: Full-time employment, part-time employment, unemployed, housewife—as a self-defined employment status—and other employment statuses, such as: Student, on sick leave, under legal working age).
(b) Obstetric variables: Number of pregnancies, including the current one, and pregnancy planning.
(c) Risk awareness of alcohol consumption during pregnancy (categorized as: (1) Risk(s) mentioned; (2) says she doesn’t know but gives an opinion; (3) she doesn’t know; (4) other answer, and the perceived duration of damage resulting from alcohol consumption during pregnancy (categorized as seven possible answers: (1) During pregnancy; (2) during childhood; (3) first years; (4) many years; (5) lifelong; (6) she doesn’t know; (7) other). The categorization of answers was performed following the piloting of the study, in which these same questions were asked, allowing those responding to provide open answers, which were subsequently categorized. Furthermore, the “other” answer option was provided, as described above.
(d) Variables related to health professionals: The numbers of health professionals who provided information and the percentage of professionals who provided correct information (recommendation not to drink any alcohol during pregnancy).
(e) Variables related to the risk perception of consuming alcohol during pregnancy for specific types of drinks (beer, wine), in terms of the amount and frequency of consumption for each of them, with five response categories: “Any amount during pregnancy is harmful”, “consuming alcohol less than once a month is not harmful”, “consuming alcohol less than once a week is not harmful”, “drinking a small amount every day is not harmful”, “drinking as much and as often as a person wants to is not harmful”. These categories were established after the answers obtained in the study pilot were studied. The “other” answer option was also included.
(f) Average daily alcohol consumption during pregnancy (in grams of pure alcohol) and average daily alcohol consumption before pregnancy (also in grams of pure alcohol). In both cases, the average daily number of grams was estimated from questions on the AUDIT scale [33], which ask about the frequency and amount of consumption of different types of drinks. Days of non-consumption were included in both calculations.

### 2.5. Data Analysis

First, a hierarchical regression analysis was conducted in order to examine the explained variance of alcohol consumption during pregnancy. In the first step, demographics (i.e., age, educational level, and employment status) were introduced in the regression equation, while obstetric history was added in the second (i.e., number of pregnancies and pregnancy planning). In the third and fourth steps, previous alcohol consumption and the partner’s alcohol consumption were included. In the fifth and sixth steps of the regression analysis, advice received from health professionals (i.e., the number of health professionals who gave advice and the percentage of health professionals who provided the correct advice) and beliefs about risks (i.e., risk awareness of alcohol consumption during pregnancy, perceived duration of the damage caused by prenatal alcohol exposure, and the risk perception of drinking beer or wine during pregnancy) were included to explain alcohol consumption during pregnancy. R^2^ were calculated at each step including subsequent variables in the analysis, as well as the change in F. Additionally, t and β coefficients were examined for each indicator. These analyses were carried out with SPSS 21.0 (IBM Corp, New York, NY, USA, 2012).

Second, moderation analyses were conducted to explore how the previous indicators (i.e., demographics, obstetric history, the partner’s alcohol consumption, advice received from health professionals, and beliefs about risks) moderate the relationship between previous alcohol consumption and consumption during pregnancy. These analyses were carried out following the recommendations described by Hayes [34], based on regression analyses. The effects described in this model represent causal assumptions, because there is no causation verification without variable manipulation. Thus, only associations among variables may be concluded. Standardized coefficients were calculated to estimate the effect of one variable (assumed to be the independent variable) on another variable (assumed to be the criterion variable), and the moderation was analyzed as the interaction between the independent variable and the moderator to explain the dependent variable. Process v3.3 macro for SPSS was used, by specifically applying the model number 1, which performs a total of 1000 bootstrap samples for bias-corrected bootstrap confidence intervals. Huber-White heteroscedasticity-consistent inference was carried out.

Third, structural equation modelling was performed to integrate the effects of demographics, obstetric history, self-reported previous alcohol consumption, the partner’s alcohol consumption, advice received from health professionals, and beliefs about risks, on self-reported alcohol consumption during pregnancy, as well as the relationships between those indicators. χ^2^, Comparative Fit Index (CFI), and Root Mean Square Error of Approximation (RMSEA) were analyzed as overall data fit indexes. Lagrange multipliers and Wald tests were sequentially performed for model modifications to improve overall fit. R^2^ was calculated to analyze the explained variance, and standardized solutions were examined. This model was tested with EQS 6.3 (Multivariate Software Inc., Temple City, CA, USA, 2017), following the recommendations by Byrne [35].

## 3. Results

### 3.1. Descriptive Characteristics of the Sample

A total of 832 pregnant women randomly selected in accordance with the procedure described above were invited to participate. Of them, 426 (51.2%) accepted. The total number of pregnant women who attended the morphological ultrasound clinic during the period in which the study was performed (5 months in 2016) was 1664. Most of the participants were Spanish (92.2%). The average age was 31.9 years (SD = 5.3). Information concerning demographic and obstetric variables was described in a previous work [32]. 

Table 1 displays the primary information from the different variables on which data were collected.

### 3.2. Regression Analysis to Explain Alcohol Consumption during Pregnancy 

Table 2 presents the results of the hierarchical regression analysis to explain self-reported alcohol consumption during pregnancy. In the first step, both educational level and employment status showed negative effects, such that lower alcohol consumption was detected in women with a higher educational level and among those self-labelled as “housewives”. In the second step, obstetric history showed a significant effect, with more alcohol consumption in women who had more pregnancies. In the third step, previous alcohol consumption had a remarkable positive effect. Those women who reported higher previous alcohol consumption also indicated higher alcohol consumption during pregnancy. In the fourth step, the partner’s alcohol consumption did not have a significant effect. In the fifth, a notable negative effect was observed for the percentage of professionals who provided correct advice. Thus, a lower percentage of professionals providing correct advice—not to consume any alcohol at all during pregnancy—is related to higher self-reported alcohol consumption. Lastly, in the sixth step, beliefs about risks were added, obtaining a final explained variance of 27%. Higher alcohol consumption during pregnancy was observed in those women who reported lower risk perception for wine. 

### 3.3. Moderation Analysis in the Relationship between Previous Alcohol Consumption and Consumption during Pregnancy

Table 3 shows the results of the regression analyses to examine moderations in the relationships between previous and current alcohol consumption. Higher alcohol consumption during pregnancy was observed in women who reported higher previous consumption and had a low level of education. With regard to obstetric history, higher consumption during pregnancy was observed in women with greater previous consumption and more experience of pregnancies. Furthermore, higher consumption was also observed in women with higher previous consumption and those who reported a lower degree of correct advice received from health professionals. Lastly, risk perception regarding beer and wine also moderated that relationship. Higher alcohol consumption during pregnancy was observed in women with higher previous consumption and lower risk perception for the two types of alcoholic drinks studied.

### 3.4. Structural Equation Model

Lastly, the relationships between the study variables were integrated in a structural equation model. After conducting Lagrange multipliers and Wald tests, the final model reached a good overall data fit, χ^2^(63, *N* = 426) = 118.17, *p* < 0.001, χ^2^/df = 1.88, CFI = 0.95, RMSEA = 0.05, 90% CI RMSEA = 0.03–0.06. Table 4 describes the effects and associations included in the model, which all reached statistical significance (*p* < 0.05). 

First, regarding alcohol consumption during pregnancy, the model showed negative effects for risk awareness of alcohol consumption during pregnancy, the percentage of health professionals who provided correct advice, educational level and employment status, with higher consumption among those women with better employment status, and positive effects for risk perception of drinking wine during pregnancy, previous alcohol consumption, and number of pregnancies. This equation presented a R^2^ = 0.25 (MSE = 0.87), with the strongest effects for the percentage of health professionals who provided correct advice and previous alcohol consumption. Second, with regard to risk perception of drinking beer during pregnancy (R^2^ = 0.05, MSE = 0.97) and wine (R^2^ = 0.01, MSE = 0.99), previous alcohol consumption was positively related. Moreover, age and the number of health professionals who provided information were negatively related to risk perception of drinking beer during pregnancy. 

Furthermore, some associations were also significant in the model. First, perceived risks of wine and beer were found to be positively interrelated. Moreover, beliefs about the duration of the damage and risk awareness of alcohol consumption during pregnancy were also positively associated. Second, the partner’s alcohol consumption was positively associated with previous consumption and age, and negatively with beliefs about the duration of the damage. Third, the number of pregnancies was negatively related to previous alcohol consumption. Moreover, pregnancy planning was positively associated with age and educational level. 

## 4. Discussion

The aim of this study was to analyze a wide range of potential predictors of alcohol consumption during pregnancy, with a view of identifying the specific weight of each of them, as well as the interactions between them. We thus intended to address the fragmented vision offered by studies conducted on this subject in general to date [8,24,31]. In order to examine the role of different factors as predictors of alcohol consumption during pregnancy, a random and representative sample of pregnant women receiving care in the outpatient clinics of a public hospital in a southern European city (Seville, Spain) was interviewed in the 20th week of pregnancy.

As in other previous studies [23,24,25,26,27], alcohol consumption prior to pregnancy was identified as the most powerful predictor of alcoholic beverage intake during pregnancy. This finding suggests, in short, that an expectant mother’s previous lifestyle tends to continue during her pregnancy, particularly in terms of products that can lead to dependence and whose consumption is socially accepted in societies like Spain, such as alcoholic beverages. In this country, the average maternal age at delivery of the first child is particularly high (31.02 years of age in 2018, [36]), and the average age to start drinking alcoholic beverages is notably low (14.1 years of age among those female students who have ever drunk alcohol, [37]), and, as such, many women will have been drinking alcohol regularly for almost 20 years before their first pregnancy. It is not easy to drastically change behavior that is deeply rooted in lifestyles and has strong social support. It should not be forgotten, furthermore, that many pregnancies are unplanned (44% worldwide [38]; 25.4% in the sample used in this study) and that alcohol may be having a teratogenic effect on the embryo before the woman is aware she is pregnant, precisely in the period of prenatal development that is most sensitive to the action of teratogens (organogenesis).

A review carried out by Stephenson et al. highlights that the state of health of the expectant mother, as well as her lifestyle during the perigestational period, has an influence on the perinatal results of the new-born baby. It therefore proves very important to promote healthy lifestyles among women of child-bearing age, and more specifically, among those who are trying to conceive, by providing preconception care [39]. According to another retrospective study, developed by Goossens et al., the adoption of these healthy lifestyles before pregnancy is more likely in nulliparous women and in those with a previous miscarriage. At the same time, women who have had previous pregnancies, as well as those who are of a lower socio-economic level are less likely to change their lifestyles and make them healthier [40]. It is therefore necessary to combine general strategies for promoting healthy lifestyles among women of child-bearing age with others aimed specifically at each sector of these women, in such a way that makes it easier for all of them to choose the healthiest options in their daily lives. 

The structural equations model created also concludes that another important predictive factor, although with less weight than the previous one, is health advice received from health professionals and, more specifically, the percentage of health professionals who have provided pregnant women with the correct advice: To abstain completely from consuming alcoholic beverages during pregnancy. This is a new finding and one that had not been previously identified in the research in this field. This result suggests that the performance of health professionals who treat pregnant women (GPs, obstetricians, midwives) may have a significant influence on their lifestyle, and specifically, on their alcohol consumption. This is consistent with the results of previous studies which corroborated the thinking that adequate health advice from professionals who care for pregnant women first-hand tends to reduce or stop alcohol consumption during pregnancy [41,42].

However, several studies carried out in the United Kingdom [43], Australia [44], and Spain [10] conclude that health advice on alcohol consumption provided to pregnant women is often contradictory and inconsistent. What the result of our study suggests is that providing pregnant women with correct advice on the issue—clearly explaining the appropriateness of not consuming any alcohol during pregnancy—proves to be crucial, as well as consistency of information in this regard among the different health professionals who care for pregnant women or women of childbearing age.

Although the predictive factors described above are those that have greater specific weight in the model developed in our work, other factors that may be relevant are also identified. Thus, the model establishes that pregnant women with better employment status (in the sense of having a full-time job) are more likely to consume alcohol than those with a part-time job or those who are unemployed. It is possible that the explanation for this lies in the likely higher level of income of the former compared to the latter. The greater their purchasing power, the more alcoholic beverages become financially accessible. A number of studies on correlates of alcohol consumption during pregnancy carried out in Ireland and Australia have concluded that high socioeconomic status (or a high income level) is associated with greater alcohol consumption during pregnancy [29,45,46]. At the same time, a study carried out in the USA also concluded that estimated level of income is positively related to drinking alcohol during pregnancy [23]. In a review of predictors of alcohol consumption during pregnancy, it was found that higher income or higher social class was found to be a predictor of drinking during pregnancy in four out of five studies that assessed this factor [25]. It has also been established that certain social situations (business dinners, etiquette…) can encourage alcohol consumption, which is a pressure that might also affect pregnant women [47].

Educational level is identified as a predictor of relevant significance in this study, with a weight equivalent to that of the number of pregnancies. The model establishes that the higher the educational level, the lower the consumption of alcohol tends to be during pregnancy. This could be interpreted as meaning that women with a higher level of education are more easily able to seek and incorporate quality information in this field. However, this result is different from that found in a study carried out with pregnant women from 15 European countries, selected through websites, in which it was detected that pregnant women and new mothers of a higher educational level are those who state in a greater proportion that they consume or have consumed alcohol during pregnancy [48]. It is possible that the relationship between the level of education and alcohol consumption during pregnancy varies depending on the country, and also, depending on the way in which the sample of pregnant women is selected, or depending on the procedure used to estimate alcohol consumption.

The model shows that a higher number of previous pregnancies has a positive effect on alcohol consumption during pregnancy. This may have a plausible explanation, since the experience of a higher number of pregnancies in pregnant women who have presumably consumed alcohol without visible negative effects in the perinatal results, can result in consumption in later pregnancies, due to decreasing the perceived risk with respect to the harmful effect of alcohol. This interpretation was described in Testa’s 1996 article [49], and later by Raymond [43], whose study suggests that pregnant women are influenced by experiences in their previous pregnancies with respect to alcohol consumption. If this consumption has not had a pernicious effect on her or on the fetus, this may make it easier for the pregnant woman to be more permissive in terms of intake.

The model identifies the perceived risk of drinking wine while pregnant as another predictor of alcohol consumption during pregnancy, in the sense that might be expected: The lower the perceived risk, the greater the consumption (estimated in grams of pure alcohol). Studies carried out in Australia [50], France [16], and Spain [32] found that low perception of the risks posed by consuming wine while pregnant dominates among expectant mothers. In Spain (and perhaps in other social contexts where low perception of the potential teratogenic effects of this alcoholic beverage dominates), it is precisely those expectant mothers who are most aware of the adverse effects on the fetus who present lower alcohol consumption during pregnancy.

Interactions between the predictors, laid out in the Results section, appear generally logical and may have clear implications for prevention. Thus, for example, it was found that the effect of alcohol consumption before pregnancy on consumption during the actual pregnancy is particularly intense when the expectant mother has a low level of education. Therefore, according to these results, those women with the lowest level of education would constitute a high-priority sector for interventions aimed at preventing FASD, probably requiring communication strategies (both within and outside the healthcare system) that are specially adapted to their socio-educational characteristics. It can also be observed that alcohol consumption by the partner and alcohol consumption before pregnancy are interrelated, which suggests that preventive interventions should not be aimed solely at expectant mothers and women before they become pregnant, but also at their partners.

As intervention suggestions resulting from the analysis of these predictive factors, we can highlight the need for health professionals to warn pregnant women of the deleterious effect of alcohol in pregnancy, conveying in clear and understandable terms the message that abstaining from alcohol consumption is the only safe practice. In turn, for health professionals to adequately assess the alcohol consumption habits of pregnant women (or women of a childbearing age in general) and develop with them culturally adapted and effective communication strategies, the implementation of suitable continued training is required, as well as the development of institutional programs in the health system that facilitate and promote the full exercising of their role in the prevention of FASD. In this field, the experience of countries that have made a greater effort in preventing FASD to date, such as Australia, Canada, the United States, and New Zealand, among others [51,52,53], should be taken into account in particular.

Finally, we should not forget that a pregnant woman is influenced by social perceptions about alcoholic beverages, as well as whatever interpretation she might have made of her own personal experiences of drinking alcoholic beverages, if she has had any. A popular perception of alcohol which has become more widespread over the last few decades is the idea that the regular consumption of moderate quantities of alcoholic beverages (wine in particular) can prove to be a cardiovascular protector. This belief serves to support another: Adults are recommended to drink low doses of alcohol regularly. Today, it is clear that both ideas are lacking in any solid foundation. According to Naimi et al. (2017), to date, there has been no randomized clinical trial of low-volume alcohol consumption that has assessed any mortality outcome. Everything we know about the impact on health of drinking low doses of alcohol is based on observational studies which, in general, suffer from serious selection biases, as detailed by these authors, which constitutes in their opinion a reason to suggest that the existing research may systematically overestimate the protective effects of “moderate” alcohol consumption [54]. At the same time, the Global Burden of Disease Study 2016, carried out using data from 195 countries and territories in the period 1990-2016, concluded that the level of alcohol consumption that minimized harm across health outcomes was zero standard drinks per week [55]. This could be expressed in other terms: No level of alcohol consumption improves health [56]. It proves easier to provide pregnant women and the people around them with correct information about the risks of drinking alcoholic beverages during pregnancy in a social context which is neutral to alcohol consumption than in one where ideas in favor of drinking are widespread. If it is explained to the population as a whole in appropriate terms by the healthcare system that there is no solid scientific basis to support the idea that moderate alcohol consumption is beneficial for health, this will contribute to reducing the enormous burden of health, social and education problems resulting from alcohol consumption, including FASD.

The study performed has both strengths and limitations. Among its strengths, it should be noted that the sample was randomly selected among all pregnant women who attended a programmed control clinic in the same week of pregnancy (week 20). Furthermore, the interviews were conducted in person (face to face) by health staff trained specifically in this area. Data analysis includes the use of a structural equations model and other confirmatory multivariate techniques. On the other hand, one limitation that can be highlighted is that, since the study is cross-sectional, it is not possible to establish inferences of causal relationships between variables. Furthermore, all immigrant pregnant women who could not take part in the interview because they had limited fluency in Spanish were excluded from the sample, due to the absence of auxiliary translation services. The participation rate was 51.2%, which does not exclude the likelihood of selection bias. Alcohol consumption was evaluated only through self-reported data, and not through biomarker analysis too. A validated scale was not used to assess the degree of planning of the pregnancy.

## 5. Conclusions

Among the conclusions derived from this study, we can establish that alcohol consumption prior to pregnancy is a predictor that has a powerful direct relationship with alcohol during pregnancy. Second, the percentage of health professionals who adequately inform pregnant women about the harmful effects of alcohol consumption in pregnancy has a powerful inverse relationship with alcohol intake during pregnancy. Furthermore, previous alcohol consumption during pregnancy is especially related to this consumption being maintained during pregnancy among expectant mothers with low educational levels.

## Figures and Tables

**Table 1:** Descriptive statistics of study variables.

Partner’s Alcohol Consumption (%)	Never = 21.4 Once a month or less = 19.5 Twice to four times a month = 29.5 Twice to three times a week = 13.3 Four or more times a week = 15.5 No partner = 0.7
Number of health professionals who provided information (%)	Zero = 43.0 One = 26.5 Two = 14.1 Three = 16.4
% of health professionals who provided correct advice	None= 19.8 A third = 0.4 Half = 2.1 Two thirds = 1.6 All = 76.1
Risk awareness of alcohol consumption during pregnancy	Risk(s) mentioned = 59.5 She doesn’t know but gives opinion = 12.7 She doesn’t know = 27.1 No risk = 0.7
Perceived duration of damage	During pregnancy = 2.4 Childbirth = 3.8 First years = 9.3 Many years = 5.2 Lifelong = 48.1 She doesn’t know = 27.5 Other = 3.8
Risk perception of drinking beer during pregnancy	Any amount is harmful = 31.5 Less than once a month is not harmful = 27.6 Less than once a week is not harmful = 25.3 A small amount every day is not harmful = 14.8 It is not harmful, regardless of the amount = 0.8
Risk perception of drinking wine during pregnancy	Any amount is harmful = 38.4 Less than once a month is not harmful = 30.6 Less than once a week is not harmful = 24.2 A small amount/day is not harmful = 5.9 It is not harmful, regardless of the amount = 0.9
Average daily alcohol consumption before pregnancy (grams)	M = 4.68, SD = 10.30
Average daily alcohol consumption during pregnancy (grams)	M = 0.38, SD = 1.55

**Table 2:** Hierarchical regression analysis of demographics, obstetric history, previous alcohol consumption, the partner’s alcohol consumption, advice received from health professionals, and beliefs about risks, as correlates of alcohol consumption during pregnancy.

Title	R^2^	ΔF	F	t	β
Step 1	0.04	5.24 **	5.24 **		
Age				−0.67	−0.04
Educational Level				−2.85	−0.14 **
Employment status				−3.91	−0.18 ***
Step 2	0.05	2.07	4.00 **		
Number of pregnancies				2.91	0.13 **
Pregnancy planning				−1.04	−0.05
Step 3	0.17	62.64 ***	14.25 ***		
Self-reported previous alcohol consumption				7.25	0.32 ***
Step 4	0.17	1.13	12.38 ***		
Partner’s alcohol consumption				1.12	0.05
Step 5	0.24	19.76 ***	14.89 ***		
Number of health professionals who provided advice				0.33	0.02
% who provided correct information				−5.29	−0.26 ***
Step 6	0.27	4.28 **	11.95 ***		
Risk awareness of alcohol consumption during pregnancy				−1.86	−0.08
Perceived duration of damage				0.02	0.01
Perceived risk of drinking beer during pregnancy				−1.85	−0.17
Perceived risk of drinking wine during pregnancy				3.18	0.29 **

*** *p* < 0.001; ** *p* < 0.01.

**Table 3:** Regression analyses of the moderations by demographics, obstetric history, the partner’s alcohol consumption, advice received from health professionals, and beliefs about risks, in the relationships between previous and current alcohol consumption.

“Previous Alcohol Consumption” x:	R^2^	F	t	β
Age	0.01	0.40	−0.63	−0.06
Educational Level	0.03	6.69 *	−2.59	−0.19 *
Employment status	0.01	1.00	1.00	0.07
Number of pregnancies	0.02	4.69 *	2.16	0.16 *
Pregnancy planning	0.01	0.03	−0.18	−0.01
Partner’s alcohol consumption	0.01	1.36	−1.17	−0.08
Number of health professionals who provided information	0.02	9.75 **	−3.12	−0.16 **
% of health professionals who provided correct information	0.10	31.94 ***	−5.65	−0.35 ***
Risk awareness of alcohol consumption during pregnancy	0.01	3.14	−1.77	−0.11
Perceived duration of the damage	0.01	2.52	1.59	0.14
Risk perception of drinking beer during pregnancy	0.02	4.43 *	2.11	0.14 *
Risk perception of drinking wine during pregnancy	0.02	4.66 *	2.16	0.15 *

Note. Dependent variable: Alcohol consumption during pregnancy. *** *p* < 0.001; ** *p* < 0.01; * *p* < 0.05.

**Table 4:** Significant effects and associations included in the structural equation model.

*Direct Effects*	
*Effects on alcohol consumption during pregnancy* -Risk awareness: −0.09 -Risk perception of drinking wine during pregnancy: 0.14 -Previous alcohol consumption: 0.33 -% who correctly informed: −0.25 -Educational level: −0.14 -Employment status: −0.18 -Number of pregnancies: 0.13	*Effects on risk perception regarding beer* -Previous alcohol consumption: 0.10 -Number of health professionals who provided information: −0.05 -Age: −0.20 *Effects on risk perception regarding wine* -Previous alcohol consumption: 0.08
*Associations*	
*Between variables:* -Partner’s alcohol consumption/previous consumption: 0.13 -Number of pregnancies/previous consumption: −0.17 -Number of health professionals who provided information/% who provided correct information: 0.37 -Employment status/% who correctly informed: −0.18 -Partner’s alcohol consumption/age: 0.12 -Partner’s alcohol consumption/perceived duration of the damage: −0.13 -Previous consumption/% who correctly informed: −0.12 -Age/educational level: 0.40 -Age/employment status: −0.23 -Number of pregnancies/age: 0.25 -Pregnancy planning/age: 0.25 -Employment status/educational level: −0.21 -Pregnancy planning/educational level: 0.20	
*Between measurement errors of the variables:* -Duration of damage/risk awareness of alcohol consumption during pregnancy: 0.32 -Risk perception regarding wine/risk perception regarding beer: 0.88	
